# Effect of Workwear Fit on Thermal Insulation: Assessment Using 3D Scanning Technology

**DOI:** 10.3390/ma18092098

**Published:** 2025-05-03

**Authors:** Magdalena Młynarczyk, Joanna Orysiak, Jarosław Jankowski

**Affiliations:** 1 The Department of Ergonomics, Central Institute for Labour Protection–National Research Institute, Czerniakowska St. 16, 00-701 Warsaw, Poland; joanna.orysiak@ciop.pl; 2 The Department of Physical Hazards, Central Institute for Labour Protection–National Research Institute, Czerniakowska St. 16, 00-701 Warsaw, Poland; jajan@ciop.pl

**Keywords:** 3D scanning, thermal insulation, air gaps, total volume

## Abstract

Thermal insulation is a basic property for describing a set of clothing and consists of the thermal resistance of the individual layers of clothing (which depends on the material used and its structure) and also takes into account the air gaps between the layers. Here, the total thermal insulation was measured in a climatic chamber with a thermal manikin. The air gaps were measured using a 3D scanning technique and calculated using the Blender 3D graphics program. Our study shows the effect of size (fit) on the size of the air gaps, as well as the influence of the air gap size on the thermal insulation value (both for static and dynamic conditions with 45 double steps and 45 double arm movements per minute) for workwear. The relationship of the total thermal insulation value on the volume and size of the air gap was described as a second-order polynomial (R2 > 0.8). It was observed that for workwear, thermal insulation did not increase when the air gaps exceeded approximately 30 mm or when the air gap volume reached 50–55 dm^3^. The highest total thermal insulation (~0.23 m^2^°C/W) was achieved when the garment closely fitted the wearer’s body (or in this case, the thermal manikin) without excessive tightness.

## 1. Introduction

**Figure 1:**
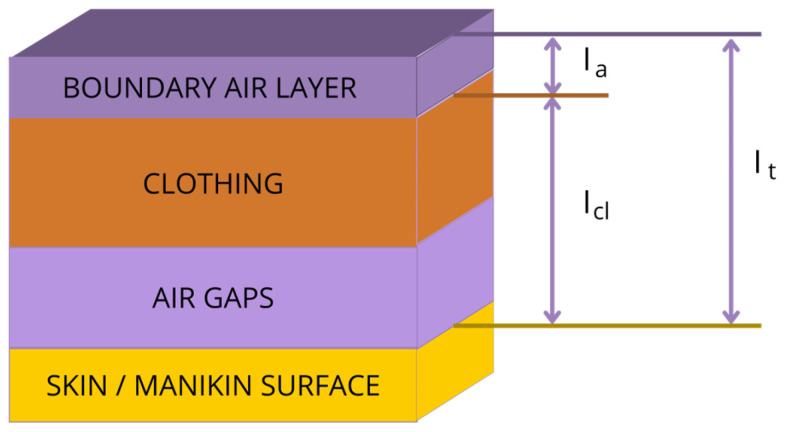
Types of thermal insulation: I_a_–thermal insulation of boundary air layer; I_cl_–basic/intrinsic thermal insulation; I_t_–total thermal insulation [2].

Heat transfer in the clothing and heat source system depends largely on the thermal insulation of the whole set of clothing. Thermal insulation is a basic property of clothing; it consists of the thermal insulation of the individual layers of clothing (which depends on the material used and its structure) as well as the air gaps between the layers [1]. According to EN ISO 9920 standard [2], several types of thermal insulation are defined, depending on which areas are taken into account (Figure 1). Total thermal insulation includes thermal insulation from the body/manikin surface to the surrounding environment, including clothing layers with enclosed air layers (air gaps) and the boundary air layer [2]. Depending on the test’s variants, two conditions were distinguished: static (on a stationary manikin, the reference condition) and dynamic (on a moving manikin with 45 double steps and 45 double arm movements per minute). The dynamic condition, therefore, takes into account the effect of activities, changes in position, and size of air gaps, as well as the “pumping effect”, where air can be exchanged via openings in clothing (such as collars, cuffs).

In the case of clothing (apart from thermal insulation), heat transfer is also influenced by parameters such as air permeability and water tightness (i.e., moisture in clothing) [3]. They result from the very structure of the garments (i.e., the materials used and the design of the garments), over which the user has no real influence. Additionally, in the clothing–heat source system, the important aspects in heat exchange are as follows: the degree of clothing fit, air movement, physical activity, body posture, and, in the case of a sitting position, the impact of the seat [4,5]. These are factors which the user can consciously influence.

Depending on the fit of the garments to the human body, air gaps are created between the layers of materials that make up the set of garments, as well as air gaps between the skin and the closest layer of material [1]. The greater the air gap, the greater the airflow underneath the garments. This has a negative impact on the thermal insulation value of the set of clothing [5]. Therefore, improperly selected underwear and outerwear that affects the formation of air gaps can significantly reduce the thermal insulation value of a set of clothing.

Heat transfer, not only in an air gap, can take place by radiation, conduction, or convection [6,7,8] and depends on the size of the air gap [7,9,10]. Thermal insulation was found to increase proportionally with the size of the air gaps, up to the point where convective heat transfer begins to occur [7,11]. Thermal conductivity and radiation are the basic methods of heat transfer in a smaller air gap, and thermal insulation increases with increasing air gap size [10,12]. In small gaps (usually less than 10 mm), thermal conduction is the dominant mechanism, and when the air gap thickness exceeds a certain threshold (usually around 10–15 mm), natural convection can occur. Ke and Wang [13], in studies of various types of clothing, showed that up to a gap of about 10 mm, thermal insulation increases because the air acts as an effective insulator. It should be emphasized that this air must be enclosed in a space (static/stagnant) [14]. Then, thermal insulation increases with the increase in the size of the air gaps [7]. It should be noted that the moisture barrier permeability has an influence on the comfort level of the clothing user [15]. In the case of firefighter clothing, both air gaps and moisture have a major impact on the thermal protection. In the study by Lu et al. [16], in air spaces < 12 mm, moisture had a stronger positive effect, whereas when the air spaces were larger (>12 mm), the effect was smaller.

However, when the air gap size is too large, due to convective heat transfer [10,17,18,19], the insulating effect of the air gap will be weakened [10,20,21,22]. According to Ke and Wang [13], in a space above 10–15 mm, natural convection occurs, which increases heat transfer and may lead to a decrease in insulation. In spaces above 37–40 mm, convection becomes the dominant mechanism, which significantly reduces thermal insulation [13].

The research conducted on heat transfer coefficients, which defined how effectively heat transfers from a manikin’s surface to the environment [23,24], has shown that they depend on, among other factors, posture (standing, sitting, or lying) and air flow velocity. It has been shown that for the whole body, under standard measurement conditions, the convective heat transfer coefficient (h_conv_) is approximately 3.3 W/m^2^°C, and the radiative heat transfer coefficient (h_rad_) is approximately 4.0–4.5 W/m^2^°C. These values signify that for every square metre of a manikin’s body surface, at a temperature difference of 1 °C, the manikin releases into the environment: 3.3 W through air movement and ~4.0 W through radiation. In total (when these two effects were added together), this value was ~7.3 W/m^2^°C. It should be noted that the lower the combined heat exchange coefficient by convection and radiation (h_eff_), the higher the thermal insulation value of the clothing. In the case of clothing for cold environments or rescue clothing, a low h_eff_ value is desirable; however, for clothing designed to effectively dissipate heat from the human body (e.g., sports clothing or for a hot environment), this indicator should be as low as possible.

In numerous publications, tests were presented on flat samples of materials, such as thin single-layer clothing or specialist clothing, e.g., for firefighters [7,14,15,25]. Heat exchange occurs differently in horizontal and vertical air spaces [26,27,28]. The position of the air spaces is significant; therefore, the use of manikins with human dimensions better reflects the position of the air spaces [29]. The aim of this study was to determine the effect of workwear fit on the thermal insulation of selected types of garments, taking into account air gaps assessed using 3D scanning technology. In the available literature, there are few reports on multi-layer clothing (e.g., [30]). Sets of clothing consisting of at least two layers were selected for this study. The tested underwear and outerwear were used by workers in the gas, fuel, and explosion hazard zone industries. These sets were deliberately selected to draw attention to the need to test such sets in order to protect employees using such clothing against radiant, convective, and contact heat, i.e., when working at a furnace, in metallurgy, or at a foundry. To demonstrate the influence of the fit to the manikin body shape on the thermal insulation value, one type of underwear (in three sizes) and four sets of outerwear (in three sizes) were used in the presented tests. The tests were conducted directly on a male-shaped thermal manikin. Using 3D scanning technology on the same thermal manikin model, both the total volume of air gaps (including between clothing layers) and the values of total thermal insulation, the resulting total thermal insulation, and the total evaporative resistance were determined.

## 2. Materials and Methods

### 2.1. Materials

One type of underwear (U) (in 3 sizes: small (S), medium (M), and large (L)) and four types of outerwear (W1–W4) (also in 3 sizes: 50, 54, and 56) were used for this study’s tests. The tested clothing items are shown in Figure 1. The selected elements of protective clothing and underwear, along with the composition of materials and possible use (according to the manufacturer’s product sheets) are presented in Table 1.

The tested underwear was used by workers in the gas, fuel, and explosion hazard zone industries and met the requirements of thematic standards (see Table 1 for normative requirements). The outerwear was used accordingly by workers of various professions in hot environments as follows: W1—construction or road service workers (professions where high-visibility clothing is required); W2—power industry, chemical industry, welding and hot environments, explosion hazard zone, high visibility; W3—welding and hot environments, explosion hazard zone, steel mills, foundries; and W4—chemical industry.

The following labels for sets of clothing were used in the study, as an example: U_S_W1_50 [underwear_/size of underwear/_set of clothing_/size of set of clothing/] denoting underwear size “S”, set of clothing “W1”, and size “50”.

The selected clothing sets were not subject to any specific requirements regarding thermal insulation values, as specified for clothing for cold environments (EN 342 [39]). In the literature, the size of air gaps and their effect on thermal insulation have been tested for specialist clothing intended for firefighters as protection against flames and burns. The selected clothing sets were intended for work in a hot environment in positions where dangerous situations such as explosions, fires, or exposure to other physical and chemical factors may occur.

### 2.2. Parameters and Methods

Since clothing for hot environments is not subject to any normative values related to thermal insulation, research procedures based on the EN 342 [39], EN ISO 15831 [40] and ASTM 2371-15 [41] standards were used to investigate the influence of air spaces on selected thermal parameters of clothing sets.

#### 2.2.1. Test Variants

Selected physical parameters (such as thermal insulation and evaporative resistance) and air gaps of clothing sets composed of one type of underwear in 3 sizes (small, medium, and large) and outerwear in 3 sizes (50—tight-fitting, 54—properly selected, and 56—too loose in relation to the thermal manikin’s figure) were tested. In total, including a naked manikin dressed in specialist skin and tests of the underwear itself, 40 test variants were performed.

#### 2.2.2. Thermal Insulation

Thermal insulation tests were carried out in accordance with the standards: EN 342 [39] and EN ISO 15831 [40] in a climatic chamber (Weiss, Buchen, Germany, type WK23’) using the Newton thermal manikin. Due to the construction of the manikin’s division into segments, there are two methods for calculating the thermal insulation value: serial determined (from the weighted surface areas of the individual segments of the manikin) and parallel averaged (for the total surface area of the manikin).

Total thermal insulation (I_t_) of the boundary air layer and each clothing variant was calculated by the serial (Equation (1)) and parallel method (Equation (3)), using the following equations [40]:

I_t_ = ∑f_i_ × [[(T_si_ − T_a_) × a_i_]/H_ci_] [m^2^K/W] (1)

f_i_ = a_i_/A (2)

I_t_ = [(T_s_ − T_a_) × A]/H_c_] [m^2^K/W] (3)

T_s_ = ∑f_i_ × T_si_ [°C] (4)

H_c_ = ∑H_ci_ [W] (5)

where T_si_—the manikin surface temperature of ith segment [°C]; T_a_—the air temperature [°C]; H_ci_—the observed dry heat loss at ith segment [W/m^2^]; a_i_—area of ith segment of manikin [m^2^]; A—total surface area of the manikin [m^2^]; f_i_—surface area coefficient; T_s_—average surface temperature of the manikin [°C]; and H_c_—total heating power delivered to the manikin [W/m^2^].

Thermal insulation is determined in static conditions (on a stationary manikin as a reference value) and in dynamic conditions (on a moving manikin as a resultant value). The dynamic condition takes into account the effect of activities, changes in position and size of air gaps, as well as the “pumping effect”, air exchanged via openings in clothing (such as collars, cuffs). The manikin then moves at a speed of 45 double steps and 45 double arm movements per minute [39]. To calculate the resultant total thermal insulation (I_tr_), Equations (1)–(5) were also used. It should be noted that tests in dynamic conditions more closely reflect real working conditions.

For selected sets of clothing, tests of dry heat exchange (thermal insulation) were carried out under static (total thermal insulation (I_t_)) and dynamic conditions (resultant total thermal insulation (I_tr_)). Thermal insulation calculations were made according to the serial and parallel model calculation [42,43,44]. At least 2 repeats were performed for each test variant.

#### 2.2.3. Evaporative Resistance

Total evaporative resistance of the clothing ensemble and surface air layer (R_et_) tests were carried out according to a modified methodology contained in the ASTM F2370-15 standard [41]. Total evaporative resistance was calculated using Equation (6) [41]:

R_et_ = [(P_s_ − P_a_) × A]/[H_e_ − (T_s_ − T_a_) × A/R_t_] [m^2^kPa/W] (6)

where P_s_—water vapor pressure at the manikin’s sweating surface [kPa]; P_a_—water vapor pressure in the air flowing over the clothing [kPa]; H_e_—power required for sweating areas [W]; A—total area of manikin’s surface that is sweating [m^2^]; and R_t_ = I_t_—total thermal resistance/insulation of the clothing ensemble and surface air layer [m^2^ °C/W]. According to the ASTM F2370 standard [41], total evaporative resistance tests can be performed under isothermal and non-isothermal conditions. It should be noted, however, that the non-isothermal variant is closer to actual operating conditions.

As part of the research, evaporative resistance was measured (based on the heat loss method [43]) in static, non-isothermal conditions with the intensity of sweating at the level of 400 mL h^−1^m^−2^. At least 2 repeats were performed for each test variant.

#### 2.2.4. Air Gap Size—3D Scanning Technique

A 3D scanner was used to determine the total air volume according to the EN ISO 20685-1 standard [45]. Each set of clothing was scanned two times.

The total volume of air gaps between the naked manikin and the outer layer of the outerwear (*V*_0_) was calculated according to the following Equation (7):

*V*_0_ = *V* − *V*_naked_manikin_ (7)

where *V*—total volume of the manikin (“lumps”, i.e., a manikin dressed in the entire set of clothes), dm^3^ and *V*_naked_manikin_—volume of a naked manikin (dressed in specialist skin), dm^3^.

The total volume of air gaps between the manikin dressed in underwear and the outer layer of the outerwear (*V*_P_) was calculated according to Equation (8):

*V*_P_ = *V*_0_ − *V*_underwear_ (8)

where *V*_underwear_—volume of manikin dressed in underwear, dm^3^.

In order to determine the average (total) size of the air spaces (A_gap_t_ [mm]) and the average size of the air gaps between underwear and the outer layer of clothing (A_gap_P_ [mm]), the following formulae were used [according to [46]]:

A_gap_t_ = *V*_0_/SA_naked_ (9)

A_gap_P_ = A_gap_t_ − A_gap_t_underwear_ (10)

where SA_naked_—the surface area of the naked manikin [1.64 m^2^]; *V*_0_—the total volume of air gaps between the naked manikin and the outer layer of the outerwear [dm^3^]; and A_gap_t_underwear_—the average size of the air gaps between the underwear and the naked manikin [mm].

### 2.3. Equipment

**Figure 2:**
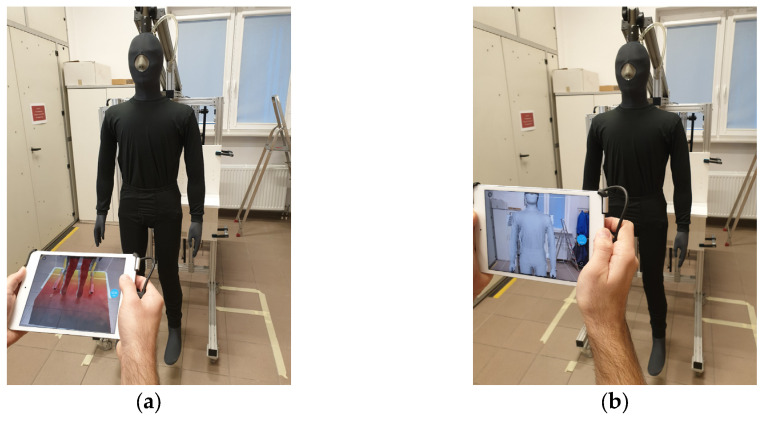
Determining the volume of the Newton thermal manikin using the following stages: (**a**) the software builds a three-dimensional mesh; (**b**) 3D scanning of the upper and lower part of manikin [48].

Physical parameters were measured using a Newton thermal manikin (Measurement Technology Northwest, Seattle, WA, USA) (Figure 2). The Newton thermal manikin, in the shape of a male body, consists of 34 segments [42,43,47,48,49]. The Newton manikin is 176 cm tall and has a chest circumference of 91 cm and a waist circumference of approx. 74 cm. The manikin has the ability to move and perform tests in dynamic conditions. The manikin can work in 3 modes: (1) constant temperature of the manikin’s surface, (2) constant flow of heat loss flux, (3) physiological mode [47]. In the presented research, the constant surface temperature mode of the manikin T_s_ = 34 °C was used to compare the obtained thermal insulation values [44].

In order to measure the thermal insulation of the clothing, the manikin was placed in the climatic chamber (Weiss, Buchen, Germany, type WK23’) [47]. The chamber has the ability to control the temperature in the range from −40 °C to 70 °C and is characterized by an air flow in the range from 0.2 m/s to 3 m/s in the horizontal direction (with the air blowing directly to the front of the manikin).

**Figure 3:**
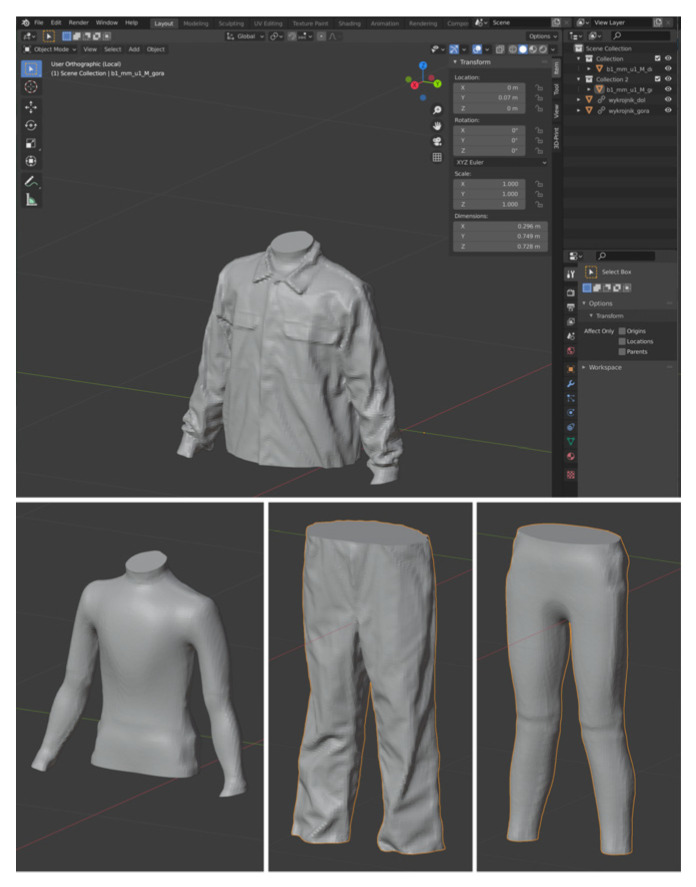
Sample calculations carried out in the Blender 3D graphics program [48].

For the 3D scanning, an iPad tablet (model number: MUQX2FD/A, software version 15.0) with a dedicated scanning adapter (MARK II sensor with model number ST02A v1.2, firmware 1.0.1) was used (Figure 1). The obtained images were computer processed using Blender 3.0 3D graphics software [48] (Figure 3).

## 3. Results

### Total Volume and Average Total Size of Air Gaps

Measured and calculated total volumes of different air gaps are shown in Table 2.

Average (total) air gap sizes and average air gap sizes between the underwear and outer garments are shown in Table 3.

**Figure 4:**
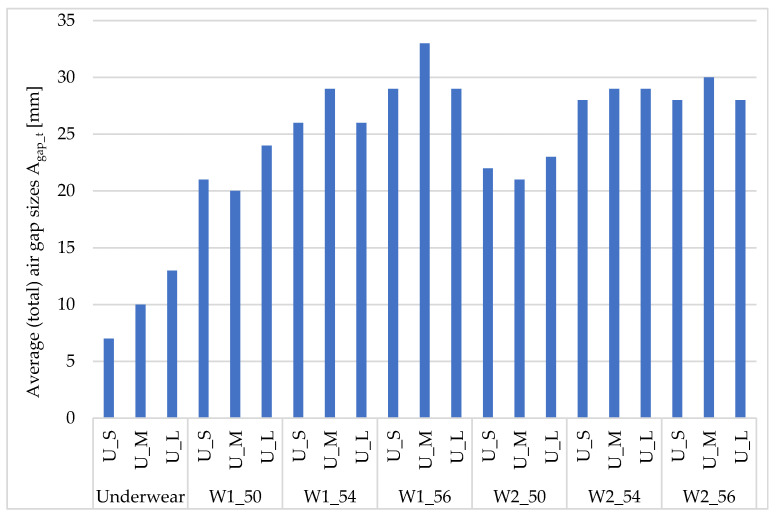
Average (total) sizes of air gaps A_gap_t_ [mm] depending on the size of underwear and outerwear (W1–W2).

**Figure 5:**
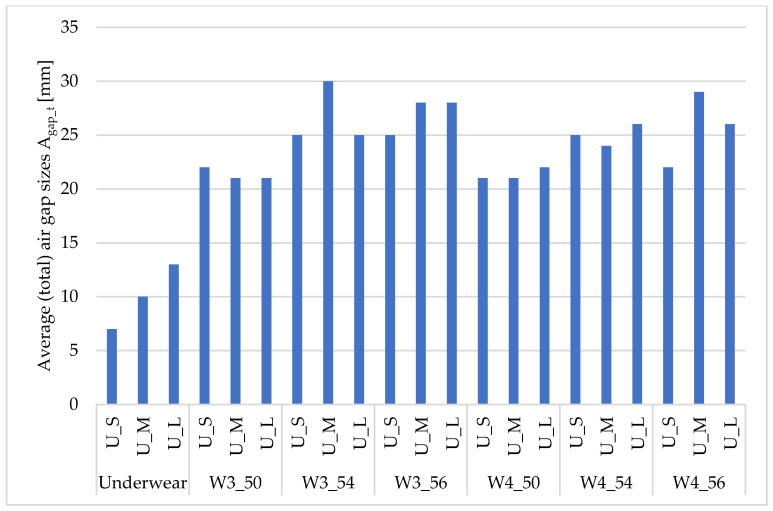
Average (total) sizes of air gaps A_gap_t_ [mm] depending on the size of underwear and outerwear (W3–W4).

Figure 4 and Figure 5 show the average (total) size of total air gaps [mm] depending on the size of the underwear and outerwear.

The calculated average (total) size of air gaps A_gap_t_ for underwear increased with its size (by approx. 3 mm) (Figure 4). In the case of the W1 clothing, the differences between the sizes of underwear (S, M, and L) for sizes 50, 54, and 56 were, respectively, 3–4 mm, 3 mm, and 4 mm. For the W2 clothing, the differences between the sizes of underwear (S, M, and L) for sizes 50, 54, and 56 were 2 mm, 1 mm, and 2 mm, respectively.

In the case of the W3 clothing, the differences between the sizes of underwear (S, M, and L) for sizes 50, 54, and 56 were 1 mm, 5 mm, and 3 mm, respectively (Figure 5). For the W4 clothing, the differences between the sizes of underwear (S, M, and L) for sizes 50, 54, and 56 were 1 mm, 1–2 mm, and 4–7 mm, respectively.

When analyzing the size of the air gaps in terms of underwear size, the smallest values were obtained for underwear sizes S and M (and outerwear size 50, regardless of the type of outerwear). For underwear in size L and outerwear size 56, similar values were obtained as for size 54. This fact can be explained by the observation that clothing of such a large size, due to its weight, adhered to the manikin in a manner similar to size 54.

**Figure 6:**
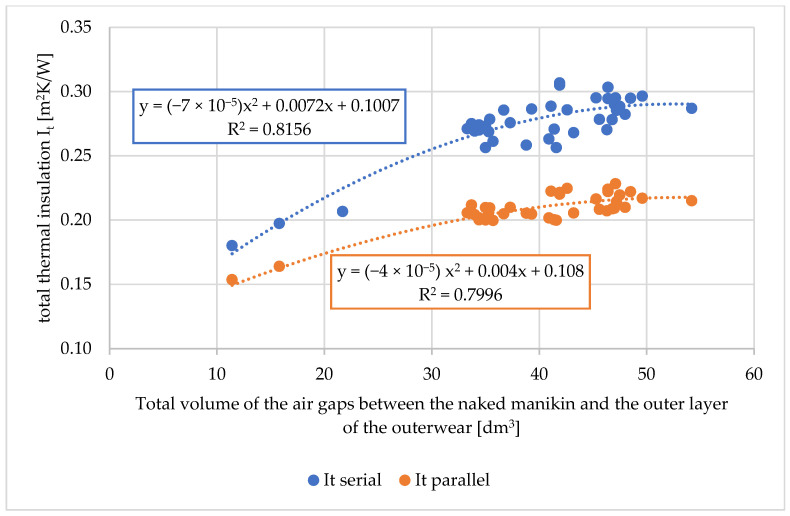
Relationship between the total thermal insulation of the tested sets of clothing (I_t_) on the total volume of air gaps between the naked manikin and the outer layer of outerwear [dm^3^].

**Figure 7:**
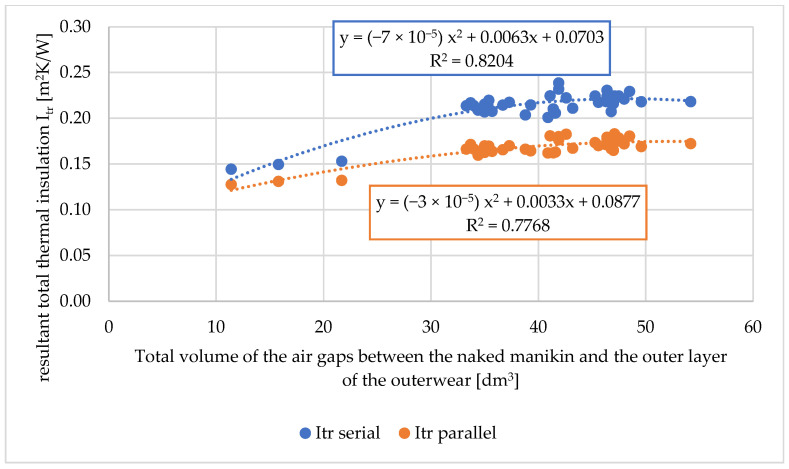
Relationship between the resultant total thermal insulation of the tested sets of clothing (I_tr_) on the total volume of air gaps between the naked manikin and the outer layer of outerwear [dm^3^].

**Figure 8:**
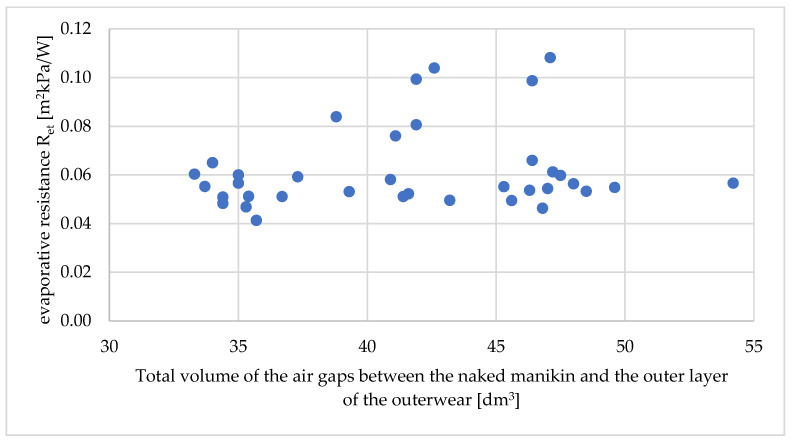
Relationship between the evaporative resistance (R_et_) on the total volume of air gaps between the naked manikin and the outer layer of outerwear *V*_0_ [dm^3^].

Based on the measured values of the total volumes of air gaps, the average (total) sizes of air gaps, the relationship of the above-mentioned values on the thermal parameters of the tested workwear were determined. The relationship between the total volumes of air gaps on the total thermal insulation, the resultant total insulation, and the water vapor resistance of the tested workwear are shown in Figure 6, Figure 7 and Figure 8.

Because the thermal manikin is divided into segments, two calculation approaches are used. The serial method tends to produce higher insulation values, as it considers the manikin as a whole, emphasizing the resistance across all segments. In contrast, the parallel method accounts for variations between segments, including those with lower thermal resistance [50]. When the tested clothing is uniform and evenly distributed across the manikin, the difference between the two methods should be low. According to Kuklane et al. [50], with an increasing number of clothing layers, the serial model value is higher relative to the parallel model. The parallel method is recommended for standard testing procedures.

For the total thermal insulation and the resultant total thermal insulation (both for the serial and parallel calculation methods), dependencies of second-order polynomials (for R2 approx. 0.8) were assigned (Figure 6 and Figure 7). Other mathematical relationships were also tested, but they were characterized by lower R2.

In the case of evaporative resistance, it was not possible to assign a mathematical relationship (Figure 8). The R2 value for the best mathematical fit (also as second-order polynomials) was approximately only 0.1.

**Figure 9:**
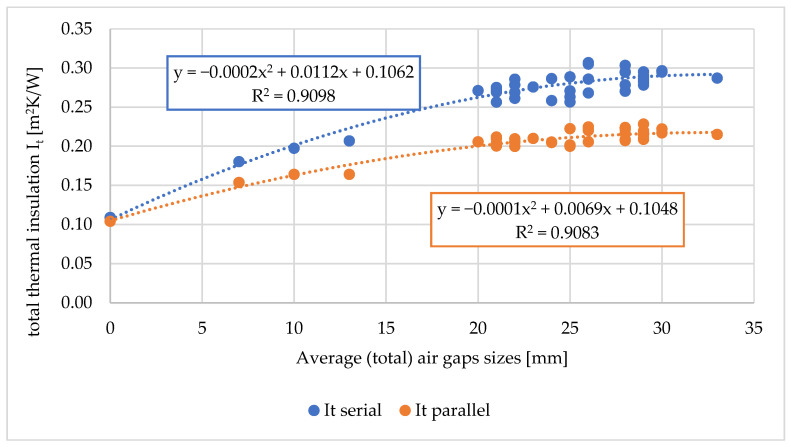
Relationship between the total thermal insulation of the tested sets of clothing (I_t_) on the average (total) size of air gaps between the naked manikin and the outer layer of outerwear [mm].

**Figure 10:**
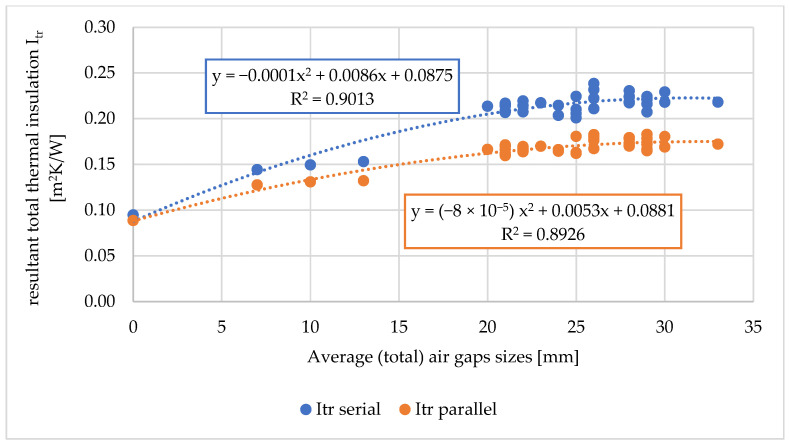
Relationship between the resulting total thermal insulation of the tested sets of clothing (I_tr_) and the average (total) size of air gaps between the naked manikin and the outer layer of outer clothing [mm].

**Figure 11:**
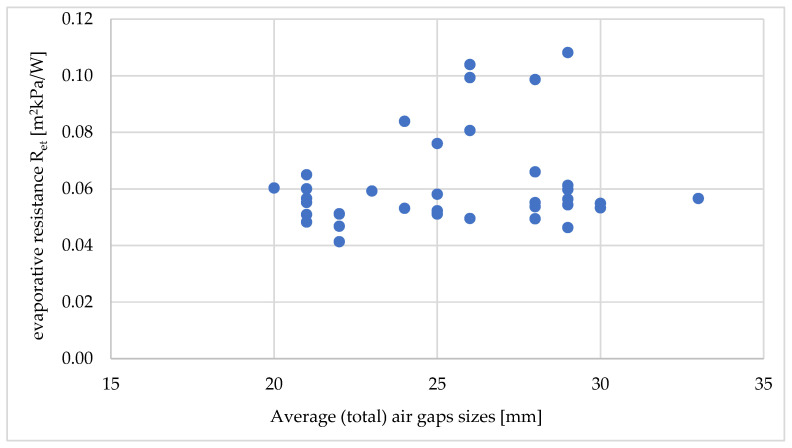
Relationship between evaporative resistance (R_et_) and the average (total) size of air gaps between the naked manikin and the outer layer of outerwear [mm].

The calculated values of the size of the air gaps were compared with the values of total thermal insulation, the resulting total insulation, and water vapor resistance, and they are presented in Figure 9, Figure 10 and Figure 11.

In the case of total thermal insulation and the resultant total thermal insulation (both for the serial and parallel calculation methods), the dependencies of second-order polynomials for (R2 approx. 0.9) were assigned (Figure 9 and Figure 10).

For the evaporative resistance, the mathematical relationships showed an R2 value of up to 0.1, which reflected a weak fit to the data (Figure 11).

In the case of the parameter thermal insulation, on the basis of the obtained mathematical relationships, the following results were determined: the total volume of air gaps and the average (total) size of air gaps of the tested sets of clothing (above which no increase in the thermal insulation value (total and resultant) was observed). The obtained results are presented in Table 4.

The volume of air gaps above which the value of thermal insulation for the tested products did not increase was 50–55 dm^3^. On the other hand, the size of the air gaps above which no increase in the thermal insulation value was observed for the tested products was 28–43 mm (Table 4).

## 4. Discussion

Depending on the fit of the garments to the human body, air gaps are created between the layers of materials that make up the tested garments, as well as spaces between the skin and the nearest material layer [1].

The study conducted by Frąckiewicz-Kaczmarek [51] showed that, under the conditions set for wearing a T-shirt, even a slight change in the size of the air gaps, e.g., by 5 mm, noticeably affected both thermal resistance and evaporation. According to the Wissler model [52], the size of the air gap influences changes in thermal resistance. With a smaller air space (<3 mm) a large amount of energy is transferred by conduction. With a larger air space, 6–7 mm or >7.5 mm, convection is initiated and the dominant form of energy transfer is a combination of radiation and convection. According to Song [7], increasing the size of the air gaps above the 7–8 mm range does not increase thermal insulation but may reduce it. For larger air spaces (>8 mm), the local heat and water vapor exchange can be increased by initiating natural convection.

The results of the tested workwear indicate that thermal insulation is affected by clothing size and, consequently, the size of the air gaps, which is consistent with the scientific literature [53,54,55]. The obtained value of thermal insulation (regardless of the type of outerwear-for four types/sectors) first increased with the size of the garments. The obtained results were consistent with the results of other authors, in which thermal insulation was better in looser clothing than in tight-fitting clothing [13,56,57,58,59]. In the case of “loose” clothing, the differences in total air gap volume between sizes 54 and 56 were smaller. The size of the air gap decreases primarily in the lower torso, depending on the fit of the garments, while remaining relatively unchanged in the upper torso. This may be due to differences in the way the clothes fall down or fit in different areas of the body [59]. The size and the shape of the air gaps had an influence on the thermal parameters [44]. The same conclusions were drawn from the presented results (3D scan images). Therefore, when analyzing the results of total air volume and air gap size, it is also important to consider the design and fit of the garments on the manikin [49].

On the basis of the performed 3D scans, the total volumes of the air gap under the clothes were determined. The air gap volume for the tested sets was within 33–54 dm^3^. The highest thermal insulation values (parallel method: I_t_~0.23 m^2^ °C/W; I_tr_~0.18 m^2^ °C/W) were obtained for the values of 47.1 dm^3^ (for total volume) and 29 mm (for average air gaps sizes). The calculated heat transfer coefficient for the I_t_ value was 4.4 W/m^2^·K. It is lower than for the naked manikin (~7.3 W/m^2^·K), which means that the clothing provides insulation from the thermal effects of the environment, limiting the heat flux reaching the body surface. This confirms its protective function in conditions of exposure to elevated temperature.

The test results were compared with the total air gap volume obtained from thermal scanning of one-piece work suits. According to Lu et al. [16], the total air volume for the tested single-layer suit ranged from 35 to 60 dm^3^. Similarly, Ke and Wang [13] examined five different multilayer ensembles, reporting a total air volume of approximately 46–74 dm^3^. These values align with the results of the present study, indirectly validating the accuracy of the measurements conducted.

Other studies show a correlation between air gap volume and thermal insulation of clothing [13,57,60]. Ke and Wang [13] also described the above-mentioned relationships as a quadratic function. After the initial increase in thermal insulation along with the increase in the size of air gaps, a decrease in insulation above a certain amount was observed [13,57,60]. The lack of a linear increase between thermal insulation and clothing size was most likely due to the fact that the air gaps reached the size/volume at which natural convection begins, and air circulates in the air gap underneath [13,57,60]. According to Epps and Song [61] and Wilson et al. [62], in the case of multi-layered clothing sets, the mathematical relationship between thermal insulation, size of air gaps, and number of layers becomes nonlinear. It was observed that natural convection occurred when the size or the volume of the air gaps were greater than 10 mm or 6 dm^3^, respectively [60]. In another study, a decrease in thermal insulation was observed when the air gap volume reached 11.9 dm^3^ [57]. In the present study, a decrease in thermal insulation was observed when the air gap volume exceeded 50–55 dm^3^ and the air gap size ranged from approximately 28–29 to 43 mm. Similarly, Ke and Wang [13] found that the threshold values for thermal insulation reduction were 55.8 dm^3^ and 37.8 mm for air volume and air gap size, respectively. However, it is important to note that those observations concerned single-layer clothing, sometimes covering only the upper or lower body [7,11,27,58,59,63,64].

The presented research, as well as examples from the available literature, indicate that the air volume under clothing could be the key parameter which determines their thermal insulation [49,59,65,66].

Taking into account the parameters, such as evaporative resistance, it was not possible to assign a satisfactory mathematical relationship (one can assign a second-order polynomial with R2~0.1). As stated in the ASTM F2370 standard [41], when tested on a thermal manikin, large air gaps in the garments system can contribute to a higher evaporative resistance than small air gaps. According to the standard, increasing air gaps may cause difficulties in the correct measurement of water vapor resistance. The inability to assign a mathematical relationship may be due to the fact that sets of different structures and materials used were analyzed. The ASTM F2370 standard [41] indicates that to compare measurements using a thermal manikin (for different materials), the clothing must be made according to the same pattern. Then, the influence of the variable design/cut and the location of the space is eliminated, since they are then kept at a constant level. Such an analysis was performed, but the relationships were determined on the basis of only three measurement points.

## 5. Conclusions

In conclusion, our study shows the effect of workwear clothing fit on the size of the air gaps, as well as the influence of the air gap size on the thermal insulation value (both for static and dynamic conditions). The relationship between the thermal insulation value on the volume and size of the air gap was described as a second-order polynomial (R2 > 0.8).

It may be concluded that for workwear, no increase in thermal insulation was observed when the air gap size reached approximately 30 mm or the air gap volume reached 50–55 dm^3^, and the best thermal insulation was achieved with a garment size that closely matched the wearer’s dimensions (in this case, to the thermal manikin’s dimensions) without overly tightening the figure.

Research also indicates the need to test multi-layer clothing and learn about the processes occurring in the individual layers of the clothing set. However, research is required not only for firefighter sets, but also for clothing designed for hot environments that protects against radiant and convective heat.

## Figures and Tables

**Table 1:** Sets of clothing consisting of underwear (U) and sets of outerwear (W1–W4).

Name	Photos of Garments	Materials Composition ^1^	Surface Mass ^1^ [gm^−2^]	Normative Requirements ^1^
U (underwear)	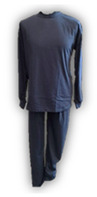	59% protex, 39% cotton, 2% negastat	205	EN ISO 13688 [31], EN ISO 11612 A1,A2,B1,C1,F1 [32], EN 1149-5 [33]
W1 (jacket and waist-length pants)	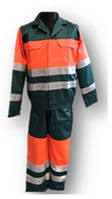	Main fabric: 80% polyester, 20% cotton Contrast fabric: 65% polyester, 35% cotton	225 245	EN ISO 13688 [31] EN ISO 20471 cl.1 [34]
W2 (jacket and waist-length pants)	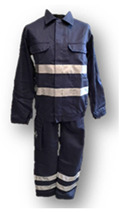	79% cotton, 20% polyester, 1% anti-electrostatic fiber; finish Hydro-Tec	260	EN ISO 13688 [31] EN ISO 11611 cl.1 A1 + A2 [35] EN 11612 A1,A2,B1,C1,E2,F1 [32] EN 1149-5 [33] EN 13034 [36] EN ISO 14116 [37] IEC 61482-2 cl.1 [38]
W3 (flame retardant jacket and flame retardant pants (waist-length)	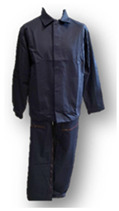	100% cotton with proban finish	335	EN ISO 13688 [31] EN ISO 11611 cl.1 A1 + A2 [35] EN ISO 11612 A1,A2,B1,C1,E2,F1 [32] EN ISO 14116 [37] IEC 61482-2 cl.1 [38]
W4 (acid-proof jacket and acid-proof dungarees)	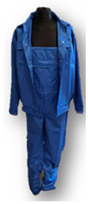	80% polyester, 20% cotton	225	EN ISO 13688 [31] EN 13034 [36]

^1^ Data from the manufacturer.

**Table 2:** Measured and calculated total volumes of the clothed manikin, the air gaps between the naked manikin and the outer layer of outerwear *V*_0_, and the air gaps between the manikin dressed in underwear and the outer layer of outerwear *V*_P_.

Study Variants	Total Volume of the Manikin *V* [dm^3^] (Scan 3D)	Total Volume of Air Gaps Between the Naked Manikin and the Outer Layer of the Outerwear *V*_0_ [dm^3^]	Total Air Gaps Volume Between the Underwear Manikin and the Outer Garments *V*_P_ [dm^3^]
naked manikin	71	-	-
U_S	82.3	11.4	-
U_S_W1_50	105.9	35.0	23.6
U_S_W1_54	114.2	43.2	31.8
U_S_W1_56	117.7	46.8	35.4
U_S_W2_50	106.2	35.3	23.9
U_S_W2_54	117.3	46.3	34.9
U_S_W2_56	116.5	45.6	34.2
U_S_W3_50	106.6	35.7	24.3
U_S_W3_54	111.9	40.9	29.5
U_S_W3_56	112.6	41.6	30.2
U_S_W4_50	105.4	34.4	23.0
U_S_W4_54	112.4	41.4	30.0
U_S_W4_56	107.6	36.7	25.3
U_M	86.8	15.8	-
U_M_W1_50	104.2	33.3	17.4
U_M_W1_54	118.1	47.2	31.4
U_M_W1_56	125.2	54.2	38.4
U_M_W2_50	104.9	34.0	18.1
U_M_W2_54	118.9	48.0	32.1
U_M_W2_56	120.6	49.6	33.8
U_M_W3_50	106.0	35.0	19.2
U_M_W3_54	119.4	48.5	32.6
U_M_W3_56	116.2	45.3	29.5
U_M_W4_50	105.4	34.4	18.6
U_M_W4_54	110.3	39.3	23.5
U_M_W4_56	118.0	47.0	31.2
U_L	92.6	21.7	-
U_L_W1_50	109.7	38.8	17.1
U_L_W1_54	113.6	42.6	21.0
U_L_W1_56	118.1	47.1	25.4
U_L_W2_50	108.3	37.3	15.6
U_L_W2_54	118.5	47.5	25.8
U_L_W2_56	117.3	46.4	24.7
U_L_W3_50	104.6	33.7	12.0
U_L_W3_54	112.0	41.1	19.4
U_L_W3_56	117.3	46.4	24.7
U_L_W4_50	106.4	35.4	13.7
U_L_W4_54	112.8	41.9	20.2
U_L_W4_56	112.8	41.9	20.2

**Table 3:** Average (total) air gap sizes (A_gap_t_) and average air gap sizes between underwear and outer garments (A_gap_P_).

Study Variants	Average (Total) Air Gap Sizes A_gap_t_ [mm]	Average Size of Air Gaps Between Underwear and Outer Garmentss A_gap_P_ [mm]
naked manikin	0	-
U_S	7	-
U_S_W1_50	21	14
U_S_W1_54	26	19
U_S_W1_56	29	22
U_S_W2_50	22	15
U_S_W2_54	28	21
U_S_W2_56	28	21
U_S_W3_50	22	15
U_S_W3_54	25	18
U_S_W3_56	25	18
U_S_W4_50	21	14
U_S_W4_54	25	18
U_S_W4_56	22	15
U_M	10	-
U_M_W1_50	20	11
U_M_W1_54	29	19
U_M_W1_56	33	23
U_M_W2_50	21	11
U_M_W2_54	29	20
U_M_W2_56	30	21
U_M_W3_50	21	12
U_M_W3_54	30	20
U_M_W3_56	28	18
U_M_W4_50	21	11
U_M_W4_54	24	14
U_M_W4_56	29	19
U_L	13	-
U_L_W1_50	24	10
U_L_W1_54	26	13
U_L_W1_56	29	16
U_L_W2_50	23	10
U_L_W2_54	29	16
U_L_W2_56	28	15
U_L_W3_50	21	7
U_L_W3_54	25	12
U_L_W3_56	28	15
U_L_W4_50	22	8
U_L_W4_54	26	12
U_L_W4_56	26	12

**Table 4:** The total volume of air gaps and the average (total) size of air gaps of the tested sets of clothing (above which no increase in the value of thermal insulation (total I_t_ and resultant I_tr_) was observed for the serial and parallel calculation method).

Study Variants	Total Air Gaps Volume [dm^3^]	Average (Total) Sizes of Air Gaps [mm]
I_t_ (serial)	51	28
I_t_ (parallel)	50	34–35
I_tr_ (serial)	45	43
I_tr_ (parallel)	55	33
I_t_ (serial)	51	28

## Data Availability

The datasets analyzed during the current study are available from the corresponding author on reasonable request.
